# Clinical Preventive Services for Patients at Risk for Cardiovascular Disease, National Ambulatory Medical Care Survey, 2005-2006

**Published:** 2011-02-15

**Authors:** Paula W. Yoon, Xin Tong, Steven M. Schmidt, Dyann Matson-Koffman

**Affiliations:** Division for Heart Disease and Stroke Prevention, Centers for Disease Control and Prevention; Centers for Disease Control and Prevention, Atlanta, Georgia; Centers for Disease Control and Prevention, Atlanta, Georgia; Centers for Disease Control and Prevention, Atlanta, Georgia

## Abstract

**Introduction:**

Clinical preventive services can detect diseases early, when they are most treatable, but these services may not be provided as recommended. Assessing the provision of services to patients at risk for cardiovascular disease (CVD) could help identify disparities and areas for improvement.

**Methods:**

We used data on patient visits (n = 21,261) from the National Ambulatory Medical Care Survey, 2005-2006, and classified patients with hypertension, hyperlipidemia, obesity, or diabetes as being at risk for CVD. We assessed differences in the provision of preventive services offered to patients who were and who were not at risk for CVD. Further, for those at risk, we compared the demographic characteristics of those who had and who had not been offered services.

**Results:**

Patients at risk for CVD received significantly more preventive services compared with those not at risk. For patients at risk for CVD, aspirin therapy was more likely to be recommended to those aged 65 years or older than those aged 45 to 64 years and to men than women. Cholesterol screening was more likely for men and was less likely for patients with Medicare/Medicaid or no insurance than for patients who were insured. Rates of counseling for diet and nutrition, weight reduction, and exercise were low overall, but younger patients received these services more than older patients did.

**Conclusion:**

Patients at risk for CVD are not all receiving the same level of preventive care, suggesting the need to clarify clinical practice guidelines and provide clinicians with education and support for more effective lifestyle counseling.

## Introduction

Death rates from cardiovascular disease (CVD) have been falling since 1980; however, several preventable risk factors are increasing (1,2). The rates of both obesity and diabetes are increasing (3,4) and may threaten the progress being made in reducing CVD mortality. Other CVD risk factors are leveling off or declining but still affect a large proportion of the US population. Data from the most recently available National Health and Nutrition Examination Survey suggest that 31% of adults (20 years or older) have hypertension and that 16% have high serum cholesterol (4). Approximately 21% of US adults smoke cigarettes (5). Preventing and managing the risk factors for CVD are an obvious point of intervention for reducing disease and premature death, yet prevention is not the mainstay of health care in the United States, and use of most clinical preventive services is low (6,7).

Clinical preventive services delivered in the primary care setting have the potential to 1) prevent risk factors for CVD, 2) detect risk factors and diseases early, when they are most treatable, and 3) treat and manage conditions before they result in serious illness or in death. The scope of services covered by the term "clinical preventive services" includes screening, testing, counseling, immunization, preventive medication, and preventive treatment. In 2005, the Centers for Disease Control and Prevention partnered with the National Business Group on Health and the Agency for Healthcare Research and Quality to develop a guide for employers that described 46 clinical preventive services that have been determined effective by authoritative organizations such as the US Preventive Services Task Force (USPSTF) (8). *A Purchaser's Guide to Clinical Preventive Services: Moving Science Into Coverage* includes descriptions of the recommended clinical services, a summary of the supporting evidence, and information about costs and delivery of the services (9). We identified 7 clinical preventive services in the guide that were recommended for the prevention of CVD: aspirin therapy; healthy diet counseling; diabetes screening; and screening, counseling, and treatment for lipid disorders, hypertension, obesity and tobacco use.

The use of these services in the primary care setting has been documented in population-based surveys (4,10,11). For example, we know from 2005 Behavioral Risk Factor Surveillance System (BRFSS) data that 62% of smokers reported that they had had 1 or more visits in the past year at which a health professional had counseled them about strategies to quit smoking (12). We also know from population-based surveys that the rates of using preventive services that involve behavioral counseling are lower than the rates for screening, immunization, or chemoprevention (13) and that there are disparities in use of preventive services by age, sex, and race (14). An alternative to using self-reports by survey participants is to examine the extent to which clinicians report providing clinical preventive services to their patients and to examine patterns in use by patient demographics.

We used data from the National Ambulatory Medical Care Survey (NAMCS) from 2005 and 2006 to assess differences in clinical preventive services offered to patients who were at risk for CVD and to those who were not at risk. For patients at risk for CVD, we also compared demographic characteristics of those who had and those who had not been offered the clinical preventive services.

## Methods

### Data sources

NAMCS is a national probability sample survey conducted annually by the National Center for Health Statistics. The survey collects data on patient visits to US nonfederal office-based physicians. The survey excludes physicians in the specialties of anesthesiology, pathology, or radiology as well as hospital outpatient departments and emergency departments. Before participating in the survey, physicians are provided with reporting forms and instructions for completing them. Each physician is randomly assigned to a 1-week reporting period when data for a systematic random sample of visits are recorded by the physician or office staff. Data are obtained on patients' symptoms, physicians' diagnoses, and medications ordered or provided. The survey also assesses the demographic characteristics of patients and services provided, including information on diagnostic procedures, patient management, and planned treatment. Further details of sampling design, estimates, and other survey information can be found at the National Center for Health Statistics' website, www.cdc.gov/nchs/ahcd.htm.

In 2005 and 2006, there were 55,057 sample patient visits to 2,526 physicians' offices, which represented an estimated 1.9 billion visits across the United States. The physicians' response rate was 62% in 2005 and 59% in 2006.

### Study population

We used data on ambulatory visits to physicians who specialized in 4 types of care: general and family practice, internal medicine, pediatrics, and cardiovascular diseases. We limited our analyses to visits described as a new problem (<3-month onset), a chronic problem (routine or flare-up), or preventive care. Visits for presurgery or postsurgery follow-up were not included. We also excluded patients who already had a diagnosis of CVD and women who were pregnant. Of 55,057 patient visits, 21,261 visits met our criteria and became the focus of this study.

### Definition of the study variables

Patients were classified as at risk for CVD if they had at least 1 of the following risk factors listed as a diagnosis related to the visit: hypertension, hyperlipidemia, diabetes, or obesity. They were also classified as at risk if the clinician indicated by check box that the patient had any of the 4 risk factors. Height and weight measurements were taken at the visit, and patients with a body mass index of at least 30 kg/m^2^ were classified as obese. Patients were considered not at risk for CVD if they did not have any of the 4 risk factors. The preventive services of interest provided at each visit were captured in 4 fields in the patient encounter form: 1) diagnostic/screening services, which included testing for glucose, hemoglobin A1c (HbA1c), and lipids/cholesterol; 2) medications (prescription and over-the-counter), including aspirin; 3) health education, including diet/nutrition, exercise, weight reduction, and tobacco use/exposure; and 4) vital signs, which included a blood pressure measurement taken at the visit. The clinical services described as healthy diet and obesity screening, counseling, and treatment in the *Purchaser's Guide* are described as diet/nutrition, exercise, and weight reduction in the NAMCS data set. Additional variables of interest that were included in the analyses were smoking status (current smoker vs other), sex, age, race/ethnicity, source of payment, and geographic region.

### Statistical methods

Two-year (2005-2006) aggregated data were used to achieve greater power for this analysis. To extrapolate our findings to national estimates, patient visit weights were used, and all estimates were related to the number of patient visits and to sample variability. Chi-square or Fisher exact tests were used to test for the significance of the associations. Multiple logistic regression analyses were conducted 1) to examine the associations between risk status for CVD (yes or no) and 8 clinical preventive services adjusting for age, sex, race/ethnicity, source of payment, region, and smoking status, and 2) among patients at risk for CVD, to examine possible associations between patient characteristics (age, sex, race/ethnicity, source of payment, region, and smoking status) and each of the clinical preventive services. All estimates derived in this analysis were based on more than 30 records, and the relative standard error was 30% or less to comply with the reliability standards established by National Center for Health Statistics. The 2-tailed *P* values were significant at <.05. All data manipulations were done with SAS version 9.1 (SAS, Inc, Cary, North Carolina), and all statistical analyses were performed with SUDAAN version 9.0 (RTI International, Research Triangle Park, North Carolina).

## Results

Of the 21,261 patient visits included in our study, 39% of visits involved patients who were classified as being at risk for CVD (Table 1). Most patients at risk for CVD had hypertension (61%), followed by obesity (44%), hyperlipidemia (41%), or diabetes (23%). Approximately 51% of patients had only 1 risk factor, 32% had 2 risk factors, 13% had 3 risk factors, and 4% had all 4 risk factors. Patients at risk for CVD differed significantly from those not at risk by age, sex, race/ethnicity, source of payment, and smoking status. Only regional variation was not significant.

Based on the physician reports, 97% of patients at risk for CVD received at least 1 of the preventive services compared with 63% of those not at risk. Multiple logistic regression analyses showed that all 8 clinical preventive services were provided significantly more often at visits of patients at risk for CVD compared with those not at risk (Table 2). Aspirin therapy was prescribed or was recommended to be continued at 6% of visits of at-risk patients compared with less than 1% for those not at risk. Screening tests were performed more often on the at-risk patients; 95% received a blood pressure check, 22% had a cholesterol test, and 18% were tested for diabetes. Education and counseling services were provided less often than screening services but were more frequent for patients at risk for CVD than for those not at risk. Of the counseling services, diet and nutrition education was offered the most frequently (26%), followed by exercise education (20%), and weight reduction education (12%). For current smokers, tobacco education was offered more often to patients at risk for CVD (34%) compared with those not at risk (25%).

To determine whether there were disparities in the preventive services offered to patients at risk for CVD, we examined the association between patient characteristics and the likelihood of services reported as provided. The likelihood of being prescribed aspirin or recommended to continue aspirin therapy was associated with age and sex (Table 3). Patients aged 65 years or older were more likely to be recommended aspirin therapy, and patients younger than 45 years were less likely, compared with patients aged 45 to 64 years. Aspirin therapy was more likely to be recommended to men than women. No significant differences by patient characteristics were detected for diabetes screening (Table 3). Cholesterol screening, however, was significantly associated with sex and source of payment. Men were more likely than women to have cholesterol screening, and patients with Medicare/Medicaid or no insurance were less likely to have cholesterol screening than patients with private health insurance. Age and region of the country were the 2 characteristics significantly associated with blood pressure screening. Patients younger than 35 years were less likely to have blood pressure screening at the heath care visit than patients aged 35 to 44 years. Patients in the Midwest, South, and West were more likely to have a blood pressure screening than patients in the Northeast.

We were unable to examine education/counseling for tobacco cessation by patient characteristics because the numbers were too small; only 34% of smokers at risk for CVD received tobacco education (Table 2). The likelihood of providing education/counseling for diet/nutrition, weight reduction, and exercise were not associated with current smoking status (Table 4). Patients younger than 20 years received significantly more diet/nutrition, weight reduction, and exercise education than did patients aged 35 to 44 years. Additionally, patients aged 45 years or older received significantly less weight-reduction education than did patients aged 35 to 44 years. Men were less likely than women to receive counseling about weight reduction and exercise, and Hispanic and "other" race/ethnicity patients were more likely to have diet/nutrition counseling than were non-Hispanic whites. Physicians were less likely to report providing exercise education to patients with Medicare/Medicaid or other sources of payment than to privately insured patients. Patients in the South received significantly less diet/nutrition education compared with patients in the Northeast, and patients in the South and West were less likely to receive weight-reduction counseling compared with those in the Northeast (half as likely for both services).

## Discussion

We found that 39% of the patients who visited physicians' offices in 2005 through 2006 who did not already have CVD were at risk for CVD, and 49% of those at risk for CVD had more than 1 of 4 risk factors. As might be expected, more clinical preventive services were provided to at-risk patients compared with patients not at risk for CVD.

At-risk patients were treated differently according to demographic and patient characteristics. Physicians were more likely to report prescribing or recommending the continuation of aspirin therapy to patients who were men and were aged 65 years or older. In 2002, USPSTF recommended that clinicians discuss aspirin therapy with adults who are at risk for coronary heart disease (CHD). Further, they suggested that the balance of benefits and risks was most favorable in patients at high risk for CHD (those with a 5-year risk ≥3%) (15). USPSTF recently revised its recommendations on aspirin use for prevention of CVD, limiting the ages to men aged 45 to 79 years and to women aged 55 to 79 years and taking both age and 10-year risk into consideration, balancing cardiovascular benefit with risk for gastrointestinal hemorrhage (16). Although the update to this recommendation was not published until 2009, providers in our study reported prescribing aspirin more frequently for men, which is more consistent with the new recommendation than with the recommendation that was current at the time of data collection.

Guidelines for when screening should start, frequency of screening, and special considerations for people at high risk for diabetes, lipid disorders, and hypertension vary according to age (17-24). Women in our study were less likely than men to receive cholesterol screening. This finding may be partly attributable to clinicians following the USPSTF guideline for lipid screening, which recommends routine screening for men from age 35 but only recommends screening for women aged 20 to 45 years who are at increased risk for CHD (19). Another guideline, the National Cholesterol Education Program Adult Treatment Expert Panel III, recommends routine blood cholesterol screening of all adults aged 20 years or older every 5 years (20). Patients with Medicare/Medicaid or with no insurance were also less likely to receive cholesterol screening than were patients with private insurance. Out-of-pocket cost to patients or differences in covered services by public-sector payers, or both, may be among the reasons for these differences. Patients younger than 35 years received blood pressure screening at their visits less often than did patients aged 35 to 44 years. USPSTF recommends that clinicians screen all adults aged 18 years or older for hypertension but does not recommend a specific screening interval (21). Many professional organizations, including the American Academy of Pediatrics and the American Heart Association, recommend that everyone aged 3 years or older have their blood pressure measured during every health care visit (24). Given that hypertension in youth is being diagnosed with increasing frequency (25) and that controlling blood pressure is one of the most cost-effective methods of reducing premature CVD (26), blood pressure screening for people of all ages should be routine.

We found lower rates of educational services for older adults (aged ≥65 y) for all 3 lifestyle interventions, although the weight-reduction counseling was the only one that was significant. Patients younger than 20 years received significantly more diet/nutrition, weight-reduction, and exercise education than did patients aged 35 to 44 years. Recommendations for lifestyle education or counseling in the clinical setting do not vary by age, although many guidelines recommend that sedentary middle-aged or older adults consult a physician before starting a new exercise program (21,22). The age discrepancy in weight-reduction education may indicate that providers believe older adults are not as willing to change behavior or are less likely to succeed at changing behavior. Older adults may also have been less likely than younger adults to be overweight or to have had more serious health problems for clinicians to address during the visit. We also found that Hispanic and other race/ethnicity groups were more likely to receive diet or nutrition education than were non-Hispanic whites and that men were less likely than women to receive weight-reduction and exercise education. Other large surveys have found that women received exercise counseling more frequently than did men (27,28). The most recent BRFSS data show that men are more likely than women to report meeting *Healthy People 2010* physical activity guidelines (52% vs 48%, respectively) (29). If men are already exercising more than women, it could account for the differences seen in exercise counseling in our study. Despite national guidelines for lifestyle counseling in the primary care setting, barriers limit its use, such as time, skills, reimbursement, coverage of services by insurance companies, and perceived effectiveness of lifestyle counseling (30). Another challenge is the multiplicity of independent guidelines from different organizations for physicians to follow. To overcome some of these barriers, health care providers can refer patients to community programs, such as wellness classes, fitness facilities, and programs offered by health plans, employers, or public health departments, for more intensive counseling (6).

This study has several limitations. First, as noted above, the data were collected per visit, not per patient. It was not possible to determine whether patients were eligible for screening tests at the visit, and most tests are not recommended at every visit. Diabetes screening, for example, is recommended only every 2 years for people at increased risk (17). Another limitation was the cross-sectional study design, which did not allow us to determine when the risk for CVD began. Additionally, the encounter form used to collect the data had little detail about the specific services provided, such as the type and intensity of the educational sessions. Lastly, since the data were reported by providers themselves or obtained from the providers' notes in the medical record, there may be some bias toward overreporting, because of either expectations or reimbursement concerns.

This study suggests that physicians are accounting for CVD risk factors and that they are providing some preventive services to most at-risk patients. However, it also identifies disparities between some subgroups in the populations of at-risk patients who are not receiving the same level of preventive care. It may be necessary to clarify practice guidelines and to specify that lifestyle interventions are appropriate and effective for all ages. Physicians also may require more education and support for effective lifestyle counseling. And finally, since the impetus for this study was *A Purchaser's Guide to Clinical Preventive Services: Moving Science Into Coverage* (9), it would be beneficial to know whether the guide is having an effect on the provision of services or on coverage for these services.

## Figures and Tables

**Table 1:** Proportion of Patients at Risk for Cardiovascular Disease, by Patient Characteristics, National Ambulatory Medical Care Survey, 2005-2006

Characteristic	No. (%), N = 21,261	At Risk for CVD,^a^ Weighted % (SE)	*P* Value^b^
Total	21,261	39.2 (1.3)	NA
**Age, y**			
<20	7,189 (34)	4.2 (0.4)	<.001
20-34	2,244 (11)	29.4 (1.7)	
35-44	2,397 (11)	44.1 (1.6)	
45-64	5,640 (27)	62.5 (1.3)	
≥65	3,791 (18)	73.3 (2.0)	
**Sex**			
Female	11,778 (55)	40.8 (1.4)	<.001
Male	9,483 (45)	37.2 (1.4)	
**Race/ethnicity**			
Non-Hispanic white	14,139 (67)	39.9 (1.6)	.003
Non-Hispanic black	2,388 (11)	43.7 (3.2)	
Hispanic	3,152 (15)	31.8 (2.1)	
Other	1,582 (7)	38.0 (3.7)	
**Source of payment**			
Private insurance	10,486 (49)	34.2 (1.5)	<.001
Medicare/Medicaid	7,414 (35)	48.8 (2.0)	
No insurance	1,131 (5)	38.0 (3.3)	
Other	2,230 (11)	35.5 (2.4)	
**Region^c^**			
Northeast	4,790 (23)	36.1 (2.2)	.17
Midwest	4,844 (23)	42.8 (3.4)	
South	6,758 (32)	40.5 (2.1)	
West	4,869 (23)	35.6 (2.4)	
**Smoking status**			
Current	2,224 (11)	50.4 (1.4)	<.001
Other	19,037 (90)	38.0 (1.4)	

Abbreviations: CVD, cardiovascular disease; SE, standard error; NA, not applicable.

a Risk defined as having hypertension, hyperlipidemia, obesity, or diabetes.

b Calculated by using χ^2^ test.

c Northeast: Connecticut, Maine, Massachusetts, New Hampshire, New Jersey, New York, Pennsylvania, Rhode Island, Vermont; Midwest: Illinois, Indiana, Iowa, Kansas, Michigan, Minnesota, Missouri, Nebraska, North Dakota, Ohio, South Dakota, Wisconsin; South: Alabama, Arkansas, Delaware, District of Columbia, Florida, Georgia, Kentucky, Louisiana, Maryland, Mississippi, North Carolina, Oklahoma, South Carolina, Tennessee, Texas, Virginia, West Virginia; West: Alaska, Arizona, California, Colorado, Hawaii, Idaho, Montana, Nevada, New Mexico, Oregon, Utah, Washington, Wyoming.

**Table 2:** Likelihood of Clinical Preventive Services Being Provided to Patients at Risk for Cardiovascular Disease Compared With Those Not at Risk, National Ambulatory Medical Care Survey, 2005-2006

Service	At Risk for CVD, Weighted % (SE)		Adjusted OR^a^ (95% CI)

	Yes	No	
Aspirin prescribed or continued	6.0 (0.7)	0.6 (0.1)	3.6 (2.6-5.0)
Diabetes screening	18.4 (1.0)	3.9 (0.4)	3.2 (2.6-4.0)
Lipid/cholesterol test	21.9 (1.0)	4.4 (0.4)	3.0 (2.5-3.7)
Blood pressure screening	95.0 (0.5)	54.6 (1.7)	3.9 (3.1-4.8)
Diet/nutrition education	26.3 (1.5)	14.9 (1.3)	4.0 (3.3-4.8)
Weight reduction education	11.6 (0.8)	1.0 (0.2)	16.9 (11.0-26.0)
Exercise education	19.7 (1.5)	7.5 (0.8)	3.3 (2.7-4.0)
Tobacco education (current smokers only)	33.7 (2.7)	24.8 (2.4)	1.5 (1.1-2.0)

Abbreviations: CVD, cardiovascular disease; SE, standard error; OR, odds ratio; CI, confidence interval.

a Data were adjusted for age, sex, race/ethnicity, source of payment, region, and smoking status except for tobacco education. Tobacco education for current smokers was assessed only after adjusting for age, sex, race/ethnicity, source of payment, and region. Eight clinical preventive services were assessed with the outcome (risk for CVD) independently by using multiple logistic regression models. All values were significant at *P* < .01.

**Table 3:** Likelihood of Clinical Preventive Services Being Provided, by Patient Characteristic for Patients at Risk for Cardiovascular Disease, National Ambulatory Medical Care Survey, 2005-2006

Characteristic	Aspirin Recommended		Diabetes Screening		Cholesterol Screening		Blood Pressure Screening	

	% (SE)	Adjusted OR^a^ (95% CI)	% (SE)	Adjusted OR^a^ (95% CI)	% (SE)	Adjusted OR^a^ (95% CI)	% (SE)	Adjusted OR^a^ (95% CI)
Total	6.0 (0.7)	NA	18.4 (1.0)	NA	21.9 (1.0)	NA	95.0 (0.5)	NA
**Age, y**								
<20	0.6 (0.5)	0.2 (0.1-0.4)^b^	12.8 (3.3)	0.7 (0.4-1.3)	13.2 (2.5)	0.6 (0.4-1.0)	74.7 (3.5)	0.4 (0.2-0.5)^b^
20-34	1.2 (0.5)		12.1 (1.7)	0.7 (0.5-1.0)	16.3 (2.0)	0.7 (0.5-1.0)	93.4 (1.5)	
35-44	1.8 (0.7)		16.9 (1.8)	1 [Reference]	21.7 (1.9)	1 [Reference]	95.3 (1.0)	1 [Reference]
45-64	5.8 (0.9)	1 [Reference]	19.6 (1.3)	1.2 (0.9-1.6)	24.0 (1.2)	1.1 (0.9-1.4)	96.1 (0.6)	1.2 (0.8-1.9)
≥65	9.4 (1.0)	2.0 (1.4-2.8)^b^	19.3 (1.4)	1.2 (0.9-1.6)	21.5 (1.4)	1.1 (0.9-1.5)	96.1 (0.6)	1.4 (0.9-2.3)
**Sex**								
Women	5.4 (0.7)	1 [Reference]	17.4 (1.2)	1 [Reference]	20.5 (1.2)	1 [Reference]	94.9 (0.6)	1 [Reference]
Men	6.8 (0.9)	1.4 (1.1-1.8)^b^	19.6 (1.2)	1.2 (0.99-1.4)	23.7 (1.2)	1.2 (1.03-1.4)^b^	95.2 (0.6)	1.1 (0.8-1.4)
**Race/ethnicity**								
Non-Hispanic white	6.3 (0.7)	1 [Reference]	18.1 (1.2)	1 [Reference]	22.6 (1.1)	1 [Reference]	95.1 (0.5)	1 [Reference]
Non-Hispanic black	4.3 (1.0)	0.8 (0.5-1.3)	17.6 (2.1)	1.1 (0.8-1.5)	19.7 (2.3)	0.9 (0.7-1.2)	96.0 (1.0)	1.3 (0.8-2.1)
Hispanic	4.2 (0.9)	0.7 (0.4-1.2)	20.3 (2.3)	1.2 (0.9-1.6)	20.6 (2.6)	1.0 (0.7-1.3)	94.1 (1.2)	0.9 (0.6-1.6)
Other	8.7 (3.7)	1.4 (0.5-3.5)	19.1 (2.9)	1.0 (0.7-1.5)	18.9 (2.8)	0.9 (0.6-1.3)	94.3 (2.9)	0.8 (0.3-2.2)
**Source of payment**								
Private insurance	5.2 (0.8)	1 [Reference]	18.3 (1.2)	1 [Reference]	23.9 (1.1)	1 [Reference]	95.1 (0.6)	1 [Reference]
Medicare/Medicaid	7.5 (0.9)	0.8 (0.5-1.1)	18.9 (1.4)	1.0 (0.8-1.3)	20.2 (1.4)	0.8 (0.6-0.98)^b^	95.2 (0.7)	0.8 (0.5-1.2)
No insurance	4.2 (0.9)	0.8 (0.5-1.3)	16.1 (2.6)	0.9 (0.6-1.3)	16.5 (3.1)	0.6 (0.4-0.99)^b^	94.1 (1.4)	0.8 (0.4-1.3)
Other			17.2 (2.4)	0.9 (0.7-1.3)	20.1 (2.4)	0.8 (0.6-1.1)		
**Region**								
Northeast	5.0 (1.5)	1 [Reference]	21.3 (2.3)	1 [Reference]	24.3 (2.0)	1 [Reference]	89.5 (1.8)	1 [Reference]
Midwest	6.3 (1.2)	1.3 (0.7-2.7)	18.8 (1.8)	0.9 (0.6-1.2)	22.4 (2.0)	0.9 (0.7-1.2)	96.1 (0.8)	3.0 (1.7-5.2)^b^
South	5.9 (1.1)	1.3 (0.6-2.6)	16.4 (1.7)	0.7 (0.5-1.1)	21.6 (1.7)	0.9 (0.7-1.2)	96.6 (0.7)	3.4 (1.9-5.9)^b^
West	6.7 (1.9)	1.3 (0.5-3.2)	19.1 (2.0)	0.9 (0.6-1.2)	19.6 (1.8)	0.8 (0.6-1.1)	95.3 (1.3)	2.6 (1.4-4.8)^b^
**Smoking status**								
Current	4.3 (0.9)	0.8 (0.5-1.2)	17.0 (1.8)	0.9 (0.7-1.2)	20.8 (1.9)	0.9 (0.7-1.1)	95.1 (1.2)	1.0 (0.6-1.5)
Other	6.2 (0.7)	1 [Reference]	18.5 (1.1)	1 [Reference]	22.0 (1.0)	1 [Reference]	95.0 (0.5)	1 [Reference]

Abbreviations: SE, standard error; OR, odds ratio; CI, confidence interval; NA, not applicable.

a The ORs for each variable were adjusted for all other variables listed in the table. The groups for age and source of payment were collapsed for modeling aspirin and blood pressure screening to comply with National Center for Health Statistics criteria.

b Significant at *P* < .05. Calculated by using χ^2^ test.

**Table 4:** Likelihood of Education and Counseling Being Provided, by Patient Characteristics for Patients at Risk for Cardiovascular Disease, National Ambulatory Medical Care Survey, 2005-2006

Characteristic	Diet/Nutrition Education		Weight-Reduction Education		Exercise Education	

	% (SE)	Adjusted OR^a^(95% CI)	% (SE)	Adjusted OR^a^(95% CI)	% (SE)	Adjusted OR^a^(95% CI)
Total	26.3 (1.5)	NA	11.6 (0.8)	NA	19.7 (1.5)	NA
**Age, y**						
<20	43.4 (4.7)	1.9 (1.2-2.8)^b^	27.4 (4.5)	1.8 (1.1-3.0)^b^	28.7 (4.3)	1.6 (1.1-2.4)^b^
20-34	26.4 (2.8)	0.9 (0.6-1.2)	18.6 (2.4)	1.1 (0.7-1.5)	21.7 (2.8)	1.1 (0.8-1.5)
35-44	28.9 (2.6)	1 [Reference]	17.4 (2.0)	1 [Reference]	20.7 (2.4)	1 [Reference]
45-64	27.1 (1.8)	0.9 (0.7-1.2)	12.0 (1.0)	0.6 (0.5-0.8)^b^	21.4 (1.8)	1.0 (0.8-1.3)
≥65	22.5 (1.9)	0.8 (0.6-1.1)	5.9 (0.7)	0.3 (0.2-0.5)^b^	15.9 (1.7)	0.9 (0.6-1.2)
**Sex**						
Women	26.8 (1.7)	1 [Reference]	12.2 (1.0)	1 [Reference]	20.4 (1.7)	1 [Reference]
Men	25.6 (1.6)	0.9 (0.8-1.0)	10.9 (0.9)	0.8 (0.6-0.99)^b^	18.7 (1.6)	0.8 (0.7-0.96)^b^
**Race/ethnicity**						
Non-Hispanic white	24.6 (1.6)	1 [Reference]	11.5 (0.9)	1 [Reference]	19.2 (1.5)	1 [Reference]
Non-Hispanic black	27.7 (2.7)	1.2 (0.9-1.6)	11.8 (1.7)	1.0 (0.7-1.3)	18.0 (2.5)	0.9 (0.7-1.3)
Hispanic	32.1 (4.5)	1.5 (1.04-2.1)^b^	12.8 (1.8)	1.1 (0.8-1.5)	23.0 (4.2)	1.2 (0.9-1.8)
Other	34.3 (3.9)	1.7 (1.1-2.5)^b^	10.2 (2.5)	1.1 (0.7-1.8)	23.3 (4.4)	1.3 (0.8-2.1)
**Source of payment**						
Private insurance	28.3 (1.8)	1 [Reference]	14.2 (1.2)	1 [Reference]	22.5 (1.7)	1 [Reference]
Medicare/Medicaid	23.2 (1.8)	0.8 (0.6-1.0)	8.1 (0.9)	0.9 (0.6-1.2)	16.4 (1.8)	0.7 (0.6-0.9)^b^
No insurance	29.8 (4.8)	1.1 (0.7-1.7)	15.2 (4.3)	1.1 (0.6-2.1)	24.6 (5.7)	1.2 (0.6-2.1)
Other	27.6 (3.3)	0.9 (0.6-1.3)	11.5 (2.1)	0.8 (0.5-1.3)	16.0 (2.6)	0.6 (0.4-0.9)^b^
**Region**						
Northeast	33.9 (3.6)	1 [Reference]	16.8 (2.2)	1 [Reference]	26.2 (3.8)	1 [Reference]
Midwest	26.4 (2.7)	0.7 (0.5-1.1)	13.3 (1.8)	0.8 (0.5-1.2)	16.9 (2.2)	0.6 (0.4-1.0)
South	22.1 (2.5)	0.6 (0.4-0.9)^b^	9.3 (1.2)	0.5 (0.3-0.8)^b^	17.7 (2.5)	0.6 (0.4-1.0)
West	27.5 (3.3)	0.7 (0.4-1.1)	9.1 (1.4)	0.5 (0.3-0.8)^b^	21.5 (3.8)	0.8 (0.4-1.4)
**Smoking status**						
Current	24.9 (2.5)	1.0 (0.8-1.2)	10.3 (1.3)	0.8 (0.6-1.1)	20.5 (2.8)	1.1 (0.8-1.5)
Other	26.5 (1.6)	1 [Reference]	11.8 (0.9)	1 [Reference]	19.6 (1.5)	1 [Reference]

Abbreviations: SE, standard error; OR, odds ratio; CI, confidence interval; NA, not applicable.

a The ORs for each variable were adjusted for all other variables listed in the table.

b Significant at *P* < .05. Calculated by using χ^2^ test.
